# Dynamic fetal cardiovascular magnetic resonance imaging using Doppler ultrasound gating

**DOI:** 10.1186/s12968-018-0440-4

**Published:** 2018-03-12

**Authors:** Fabian Kording, Jin Yamamura, Manuela Tavares de Sousa, Christian Ruprecht, Erik Hedström, Anthony H. Aletras, P. Ellen Grant, Andrew J. Powell, Kai Fehrs, Gerhard Adam, Hendrik Kooijman, Bjoern P. Schoennagel

**Affiliations:** 1 Department of Diagnostic and Interventional Radiology and Nuclear Medicine, University Medical Center Hamburg-Eppendorf, Martinistraße 52, 20246 Hamburg, Germany; 2 Department of Obstetrics and Fetal Medicine, University Medical Center Hamburg-Eppendorf, Martinistraße 52, 20246 Hamburg, Germany; 3 Department of Clinical Sciences Lund, Clinical Physiology, Lund University, Skane University Hospital, Lund, Sweden; 4 Department of Clinical Sciences Lund, Diagnostic Radiology, Lund University, Skane University Hospital, Lund, Sweden; 5 Laboratory of Computing, Medical Informatics and Biomedical-Imaging Technologies, Aristotle University of Thessaloniki, School of Medicine, Thessaloniki, Greece; 6 Departments of Radiology and Medicine, Boston Children’s Hospital, and Harvard Medical School, Boston, MA USA; 7 Department of Cardiology and Department of Pediatrics, Boston Children’s Hospital, Harvard Medical School, Boston, MA USA; 8 Philips GmbH, Röntgenstrasse 22, 22335 Hamburg, Germany

**Keywords:** Cine MRI, Fetal heart, Cardiovascular magnetic resonance imaging, Doppler ultrasound, Cardiac-gated imaging techniques

## Abstract

**Background:**

Fetal cardiovascular magnetic resonance (CMR) imaging may provide a valuable adjunct to fetal echocardiography in the evaluation of congenital cardiovascular pathologies. However, dynamic fetal CMR is difficult due to the lack of direct in-utero cardiac gating. The aim of this study was to investigate the effectiveness of a newly developed Doppler ultrasound (DUS) device in humans for fetal CMR gating.

**Methods:**

Fifteen fetuses (gestational age 30–39 weeks) were examined using 1.5 T CMR scanners at three different imaging sites. A newly developed CMR-compatible DUS device was used to generate gating signals from fetal cardiac motion. Gated dynamic balanced steady-state free precession images were acquired in 4-chamber and short-axis cardiac views. Gating signals during data acquisition were analyzed with respect to trigger variability and sensitivity. Image quality was assessed by measuring endocardial blurring (EB) and by image evaluation using a 4-point scale. Left ventricular (LV) volumetry was performed using the single-plane ellipsoid model.

**Results:**

Gating signals from the fetal heart were detected with a variability of 26 ± 22 ms and a sensitivity of trigger detection of 96 ± 4%. EB was 2.9 ± 0.6 pixels (4-chamber) and 2.5 ± 0.1 pixels (short axis). Image quality scores were 3.6 ± 0.6 (overall), 3.4 ± 0.7 (mitral valve), 3.4 ± 0.7 (foramen ovale), 3.6 ± 0.7 (atrial septum), 3.7 ± 0.5 (papillary muscles), 3.8 ± 0.4 (differentiation myocardium/lumen), 3.7 ± 0.5 (differentiation myocardium/lung), and 3.9 ± 0.4 (systolic myocardial thickening). Inter-observer agreement for the scores was moderate to very good (kappa 0.57–0.84) for all structures. LV volumetry revealed mean values of 2.8 ± 1.2 ml (end-diastolic volume), 0.9 ± 0.4 ml (end systolic volume), 1.9 ± 0.8 ml (stroke volume), and 69.1 ± 8.4% (ejection fraction).

**Conclusion:**

High-quality dynamic fetal CMR was successfully performed using a newly developed DUS device for direct fetal cardiac gating. This technique has the potential to improve the utility of fetal CMR in the evaluation of congenital pathologies.

## Background

Fetal magnetic resonance imaging is an increasingly used diagnostic tool for evaluation of the fetal central nervous system, thorax, and abdomen [1–3]. In contrast to other organ systems, a comprehensive assessment of the fetal cardiovascular system requires dynamic imaging to resolve cardiac motion and blood flow. However, the clinical application of fetal cardiovascular magnetic resonance (CMR) imaging lags behind due to technical challenges [4]. The main challenge is the lack of a cardiac gating signal that synchronizes image data acquisition to the cardiac cycle and builds images over multiple cardiac cycles to optimize temporospatial resolution. Cardiac gating is the main technique used post-natally for dynamic CMR and utilizes the patient’s surface electrocardiogram (ECG) signal to track the cardiac cycle. The fetal ECG signal, however, cannot be obtained in-utero during CMR.

Doppler ultrasound (DUS) is a well-established technique that allows real-time assessment of the fetal heart rate [5]. As DUS is theoretically not influenced by the electromagnetic field of the CMR scanner [6, 7], it provides an opportunity to synchronize CMR data acquisition with the fetal cardiac cycle. The recent innovation of a CMR compatible DUS transducer demonstrated promising results to overcome the shortcomings of fetal cardiac gating. The DUS gating technique was applied for cardiac cine imaging in healthy adult human volunteers, revealing excellent agreement of quantitative and qualitative parameters compared to ECG and pulse oximetry gating methods [8, 9]. Initial trials demonstrated the potential of the DUS transducer for gating also of the fetal heart performing cardiac cine imaging and phase-contrast CMR angiography in sheep fetuses [10, 11]. However, the transfer of this gating method to human fetuses is associated with different imaging conditions. Unlike the previous feasibility studies in sedated and intubated animals and healthy adult humans, CMR imaging of pregnant women is much more challenging. Examination time may be limited due to comfort reasons of the pregnant women, and breath-hold duration may also be limited due to pregnancy. The human fetus is also more likely to move during the scan as compared with fetuses in sedated animal.

Fetal cardiac gating using an external gating device has not yet been evaluated in human fetuses. Therefore, the aim of this study was to investigate the effectiveness of a newly developed CMR compatible DUS device for cardiac gating in the human fetus.

## Methods

### Study subjects

The CMR compatible DUS device was evaluated prospectively at three centers: University Medical Center Hamburg-Eppendorf, Hamburg, Germany; Boston Children’s Hospital, Boston, Massachusetts, United States; and Lund University Hospital, Scania, Sweden. A total of 15 pregnant women at 30–39 weeks gestation participated in the study. Five of them were enrolled following fetal ultrasound that raised concern for congenital malformation. The remaining 10 subjects were recruited as part of a multi-center study of fetal CMR. The study was approved by the respective local ethic committees, and written informed consent was obtained from all participants.

### Cardiac gating

**Fig. 1:**
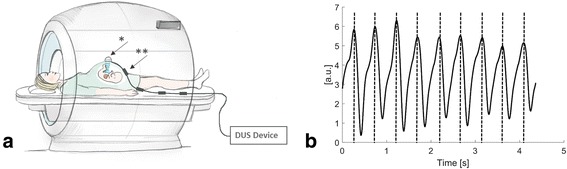
Doppler Ultrasound Trigger Device. **a** Schematic illustration of the experimental setup during fetal CMR showing placement of the Doppler ultrasound (DUS) transducer (*) on the maternal abdomen. The connecting cable has four traps (**) to avoid electromagnetic interferences from radiofrequency pulses. **b** Example of generated DUS gating signals represented by maximum signal peaks. The fetal heart beat was recorded by the DUS transducer and processed to allow for maximum peak detection

A newly developed DUS device was used for external fetal cardiac gating (northh medical GmbH, Hamburg, Germany) following the principles as previously described for cardiac gating of the adult heart (Fig. 1a) [9]. A commercially available DUS transducer (HP 15245A, Hewlett Packard, Palo Alto, California, USA) was made CMR compatible by replacing all magnetic components with non-magnetic materials. The DUS device was designed to transmit 1.024 MHz ultrasound pulses at a repetition frequency of 3.2 kHz and an acoustic intensity of 4.6 mW/cm^2^ to the connected DUS transducer inside the CMR bore. The DUS transducer was connected to the DUS device using a 7 m long transmission line. Electromagnetic interferences between the radiofrequency (RF) field of the CMR scanner and the transmission line were minimized by using four cable traps located 30 cm apart and tuned to 64 MHz [12]. The DUS signal received by the transducer is mainly based on fetal cardiac wall motion [13]. The less prominent effect of blood flow was neglected by low-pass filtering with a cut-off frequency of 100 Hz. The recorded signal (0–10 Hz) from the DUS transducer underwent peak detection analysis implemented on a microcontroller (STM32F4, STMicroelectronics, Geneva, Switzerland) that was part of the DUS device, processing data in real time. The resulting fetal cardiac DUS signal used for trigger detection is shown in Fig. 1b. Dedicated software estimated the RR interval by autocorrelation of the first 1.250 ms of the recorded signal as previously described [9]. A shiftable cover window referring to the estimated RR interval and previous detected trigger time points enabled calculation of the next expected trigger time point and hence allowed exclusion of peaks related to motion during myocardial relaxation. To provide a cardiac gating signal for the MR scanner, the external input terminal for gating signals was used which requires a normed Transistor-transistor-logic (TTL) signal. A standard Bayonet Neill Concelman (BNC) connection was used to transfer the calculated gating signals from the DUS device to the external input terminal of the CMR scanner.

The generated DUS gating signals were stored for each fetus to assess parameters such as heart rate variability and sensitivity of trigger signal detection. The sensitivity of trigger signal detection was calculated using visually defined fetal heart beats from the original DUS signal as an external reference.

### Image acquisition

All scans were performed on clinical 1.5 T CMR scanners (Philips Achieva, Best, the Netherlands at University Medical Center Hamburg-Eppendorf and Boston Children’s Hospital; Siemens Avanto, Siemens Healthineers, Erlangen, Germany at Lund University Hospital) using a multichannel cardiac coil. Examinations were performed with the subjects in a left decubital or supine position. First, the position of the fetus was palpated. Subsequently, the DUS transducer was moved over the lower maternal abdomen above the fetal thorax until a constant fetal cardiac signal was recorded. The transducer was then held in position by an elastic belt around the maternal abdomen. The coil was placed external to the transducer.

Real-time interactive balanced steady-state free precession (bSSFP) imaging was used to plan cardiac imaging planes. Using the fetal cardiac gating signal from the DUS device, retrospectively gated bSSFP cine imaging (Table 1) was performed in cardiac 4-chamber and short-axis views during maternal breath-hold.

**Table 1:** Scan parameters of DUS-gated balanced SSFP cine sequences at different imaging sites

	University Medical Center Hamburg-Eppendorf	Boston Children’s Hospital	Lund University Hospital
TR/TE [ms]	3.3 / 1.6	3 / 2	37 / 1.4
Flip Angle [°]	60	60	56
Parallel Reduction Factor	2	1.5	2
Heart Phases	20	20	30
Temporal Resolution [ms]	24	20	14
FOV [mm]	300 × 300	280 × 280	329 × 360
Matrix Size	288 × 288	320 × 320	234 × 256
Pixel Spacing [mm]	1.04 × 1.04	0.87 × 0.87	1.4 × 1.4
Number of Slices	1	1	1
SliceThickness [mm]	5	4	4
Scan Length [s]	8	8	9

*FOV* field of view, *TE* echo time, *TR* repetition time

The DUS signal and the corresponding gating signal were stored during CMR image acquisition and later evaluated to determine the quality of the gating signal in terms of heart rate variability and sensitivity to detect each heartbeat.

### Image analysis

Qualitative analysis consisted of independent image quality ratings by two radiologists (7 and 12 years CMR experience). The evaluation process included two different aspects: 1) overall image quality and 2) cardiac diagnostic quality by structure visualization. The grading system was based on a 4-point scale [14].

*Overall image quality*: 1 = low quality and/or high degree of artifacts, 2 = moderate quality and/or some artifacts, 3 = high quality and/or few artifacts, 4 = high quality and no artifacts.

*Cardiac diagnostic quality*: 1 = inadequate, 2 = low, 3 = moderate, 4 = high.

The basis for evaluation of diagnostic quality included discernible epi- and endocardial borders to surrounding fetal lung and ventricular lumen, respectively, visualized papillary muscles / trabeculation, delineation of the atrial septum and the foramen ovale, and visualized myocardial wall thickening during contraction in the 4-chamber view.

Quantitative analysis of DUS-gated images was assessed using the edge contrast between myocardium and lumen [15]. This method uses the edge spread function of myocardial and ventricular signal intensities and allows calculation of endocardial border sharpness (EBS). The edge spread function describes the contrast of an image in terms of the gradual transition (i.e. from black over gray to white), averaging all pixels in the vertical direction and parallel to the edge [16]. For optimal image contrast two pixels with different signals are required, where the slope of the edge spread function would be equal to one. Inaccurate synchronization of the cardiac cycle is the major cause for image blurring, resulting in a decreased slope of the edge spread function, and higher number of pixels, respectively. To assess an EBS impaired by motion blurring the slope of the edge spread function between myocardium and lumen was calculated as

$$ EBS=\max \left(\frac{dS(r)}{dr}\right) $$

where S(r) is the edge spread function. Prior to calculation, S(r) was normalized using the mean lumen signal intensities and a baseline corrected by subtracting S(r) from the mean myocardial signal intensities. Hence, 1/EBS determines the width of pixels between mean myocardial signal intensities and lumen, describing endocardial blurring (EB). An EB value of 2 (pixels) is the theoretical optimum image contrast to differentiate the myocardium from the lumen, whereas higher values reflect increased motion artifacts and hence image blurring. EB was calculated in a slice perpendicular to the mid-ventricular myocardium for the 4-chamber and short-axis views, respectively, using a dedicated algorithm in Matlab (The Mathworks, Natick, Massachusetts, USA). For analysis in short-axis views, the ventricle was segmented into 36 radial sections. EB was then calculated for each heart phase and section over a whole RR interval. End-systolic and end-diastolic EB were calculated as specific time points.

Quantitative analysis also included left ventricular (LV) volumetric data and measurements of the myocardial wall thickness in end-diastole. LV volumetry was calculated using the single plane ellipsoid model [(8 x A^2^ / (3 x π x L)], where A is the LV 4-chamber area and L the long-axis length (mitral valve plane to the apex) in the 4-chamber view.

All quantitative measurements were assessed by the same observers as above. LV end-diastolic volume (EDV), end-systolic volume (ESV), stroke volume (SV) and ejection fraction (EF) were determined by manual delineation of the endocardial border and the mitral valve plane in end-diastolic and end-systolic 4-chamber views using a dedicated DICOM viewer software (OsiriX Lite, Pixmeo, Bernex, Switzerland).

### Statistical analysis

Numerical data are given as mean (± standard deviation, SD). Differences between groups were analyzed using Students t-test with significant differences defined by *p* < 0.05. Quantitative cardiac measurements were compared between two observers by Bland-Altman plots using a statistical EXCEL add-on software package (Analyze-it Software, Ltd., Leeds, UK) within 95% limits of agreement. A linearly weighted 4 × 2 Kappa test was used to assess the inter-observer agreement for image quality scores.

## Results

### Examination yield

Among the 15 subjects who underwent DUS gated fetal CMR, 4-chamber views were obtained in all and short-axis views were obtained in 11. In the remaining 4 cases, the CMR examination was terminated because of patient discomfort related to the small confines of the scanner bore. No subject reported discomfort in the region of the DUS transducer.

### DUS signal quality

In all cases the DUS device produced a fetal cardiac motion signal. In four cases, the signal was lost during the examination and was regained with repositioning the DUS transducer; this transient loss of signal was attributed to major fetal movement based on review of the localizing images. In some cases, there was loss of the DUS gating signal when the patient held her breath for imaging acquisition; the signal returned when breath-holding was completed. Loss of the DUS gating signal may occur if the fetal heart moves out of the acoustic window of the DUS transducer. This may be caused by deep maternal inspiration which can result in movement of the maternal abdominal organs, and also the fetus. In such cases, the women were asked to use a shallower inspiration for breath-holding, solving the issue. In 7 cases where the examination also included other fetal CMR imaging, efficient scanning without need of repositioning the DUS transducer was up to 45 min. No distortion of the DUS gating signal from electromagnetic interferences was observed during image acquisition. Fetal heart rates varied within normal ranges (130 to 163 bpm).

Analysis of the DUS gating signal during CMR image acquisition resulted in a mean heart rate variability of 26 ± 22 ms. The evaluated gating signals were the sum of gating signals used for acquisition of cine images in 4-chamber and short-axis views per fetus. The examination time and thus the number of evaluated gating signals per fetus varied especially due to incomplete examinations in single fetuses as mentioned above. The mean number of gating signals employed for all performed cine acquisitions were 64 ± 21 per fetus, where 2 ± 1 trigger were missed or not detected, resulting in a mean sensitivity to detect each fetal heartbeat of 96 ± 4%. Results of the gating signal used for image acquisition are shown for each subject in Table 2.

**Table 2:** DUS Gating characteristics

Subject	RR Interval [ms]	Variability [ms]	RR Rejected	Trigger^a^	Sensitivity [%]
1	461 ± 25	26 ± 22	0	72	100
2	442 ± 42	33 ± 23	3	36	92
3	445 ± 32	43 ± 29	0	82	100
4	368 ± 21	44 ± 43	4	74	95
5	394 ± 14	38 ± 28	6	86	93
6	399 ± 18	20 ± 25	3	58	95
7	428 ± 25	31 ± 21	0	25	100
8	382 ± 12	17 ± 13	1	97	99
9	388 ± 12	19 ± 15	0	34	100
10	397 ± 14	28 ± 31	0	85	100
11	400 ± 18	13 ± 12	4	71	94
12	422 ± 29	12 ± 9	2	61	97
13	446 ± 41	17 ± 24	4	90	96
14	400 ± 41	18 ± 18	2	78	97
15	454 ± 27	31 ± 32	2	76	97
Mean	418 ± 25	26 ± 22	2 ± 1	64 ± 21	97 ± 4

^a^Number of trigger signals vary dependent on all acquired cine images per fetus

### Qualitative evaluation of overall image quality and cardiac diagnostic quality

**Fig. 2:**
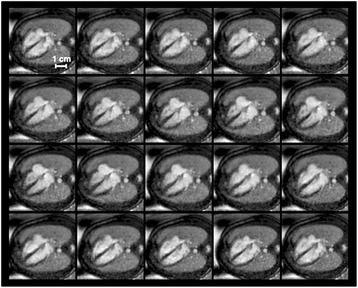
Multiple Phases of DUS Gated Cine Images. DUS-gated balanced SSFP cine images (20 phases) of the fetal heart in the 4-chamber view (gestational week 36). DUS gating allowed for clear differentiation of the myocardium versus lumen throughout the cardiac cycle. Symmetric contraction of the ventricles and expansion of the atria is seen with maximum ventricular contraction and minimum ventricular blood volume in cardiac phases 10–12

All fetal cardiac cine images gated with the external DUS device could be used for analysis. Successful fetal cardiac gating was demonstrated by clear demarcation of the myocardium from surrounding structures over the whole cardiac cycle for all fetuses (Fig. 2). Overall image quality was high with no or only few artifacts (3.6 ± 0.6). Agreement in overall image quality between the two observers was good (kappa = 0.67 ± 0.12).

**Fig. 3:**
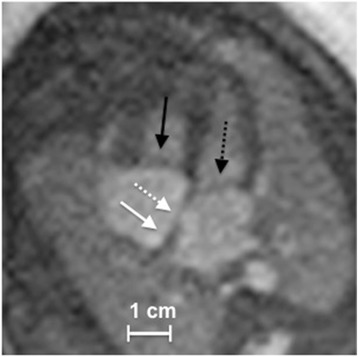
Example of DUS Gated Cine Image. DUS-gated bSSFP cine image of a fetus (gestational week 34) in the end-systolic 4-chamber view demonstrating the foramen ovale (dashed white arrow), the atrial septum (white arrow), the mitral valve (dashed black arrow) and the tricuspid valve (black arrow)

**Fig. 4:**
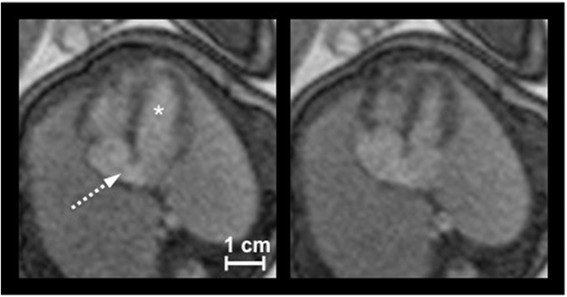
DUS Gated Cine Image in End-Diastole and End-Systole. DUS-gated bSSFP 4-chamber cine views of a fetus (gestational week 35) in end-diastole (left) and end-systole (right) illustrating the foramen ovale (white dashed arrow) as a discontinuity of the atrial septum (* = left ventricle). Images demonstrate clear differentiation of the hypointense myocardium from the hyperintense ventricular blood and lung, respectively. Myocardial thickening can be noticed in end-systolic images

Evaluation of cardiac diagnostic quality by structure visualization according to the 4-point scale (1 = inadequate, 2 = low, 3 = moderate, 4 = high) was moderate to high with mean values of 3.4 (± 0.7) for the mitral valve, 3.4 (± 0.7) for the foramen ovale, 3.6 (± 0.7) for the atrial septum, 3.7 (± 0.5) for the papillary muscles, 3.8 (± 0.4) for the differentiation of myocardium/lumen, 3.7 (± 0.5) for differentiation of myocardium/lung, and 3.9 (± 0.4) for myocardial thickening during systole. Examples of gated 4-chamber cine images illustrating the evaluated cardiac structures are shown in Fig. 3 and Fig. 4**.** Inter-observer analysis revealed good to very good agreement for visualization of the mitral valve, foramen ovale, atrial septum, myocardial thickening, and myocardium/lung interface (kappa = 0.63–0.84) and moderate agreement for papillary muscles and myocardium/blood interface (kappa = 0.57 and 0.58).

### Endocardial blurring and LV volumetry

**Fig. 5:**
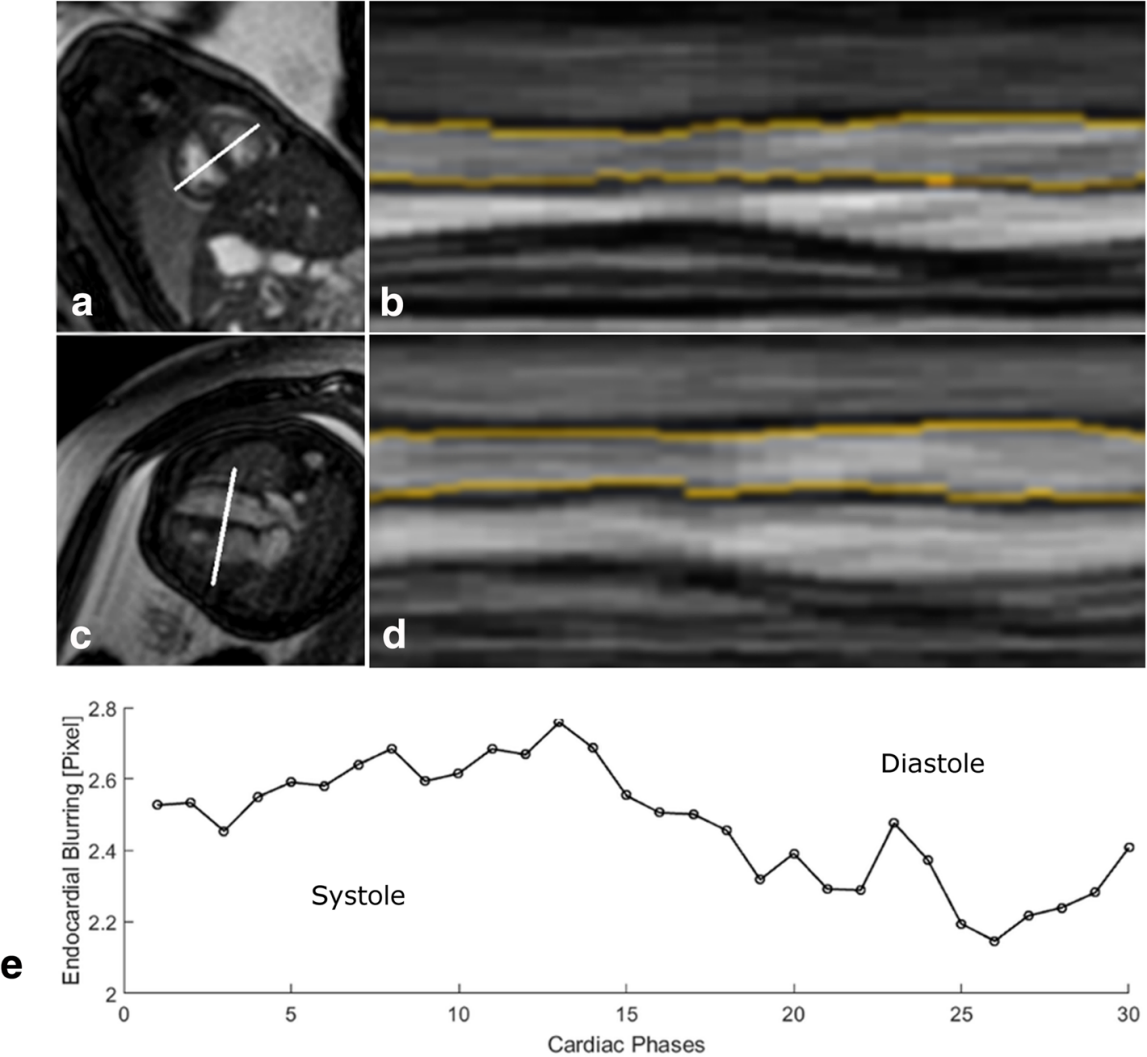
Left Ventricular Myocardial Wall Motion and Endocardial Blurring. Left ventricular (LV) wall motion and endocardial blurring for DUS-gated fetal CMR. Mid-ventricular short-axis and 4-chamber images in end-diastole are shown in **a**) and **c**) with the solid line marking the plane for the corresponding projection of myocardial wall motion over the cardiac cycle as shown in **b**) and **d**) with the endocardial border marked orange. Average endocardial blurring of LV wall motion over the entire cardiac cycle is shown in **e**) and was sensitive to cardiac phases in terms of reduced EB during diastole

Endocardial blurring (EB) due to motion was sensitive to the cardiac cycle with different mean end-diastolic (2.4 ± 0.1 pixel) and end-systolic EB (2.6 ± 0.1 pixel) (*p* < 0.001) (Fig. 5). Mean EB over the entire cardiac cycle was 2.9 ± 0.6 pixel for images in 4-chamber views assessed in 15 fetuses and 2.5 ± 0.1 pixel for images in short axis views assessed in 11 fetuses.

Fetal LV volumetry was performed in all subjects with LV mean values of 2.8 ± 1.2 ml (EDV), 0.9 ± 0.4 ml (ESV), 1.9 ± 0.8 ml (SV), and 69.1 ± 8.4% (EF), respectively. Myocardial wall thickness was assessed with 3.4 (± 0.5) mm.

**Fig. 6:**
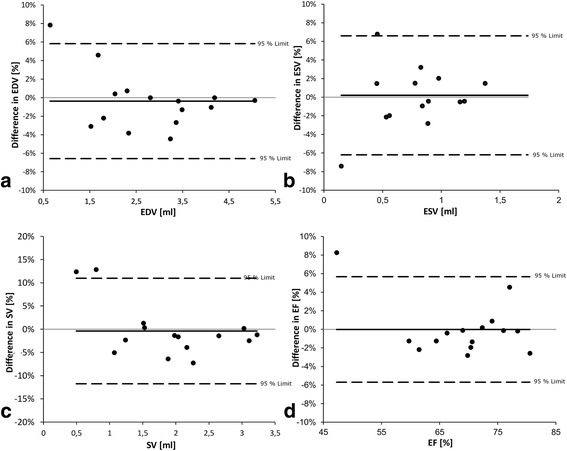
Bland Altman Plots for Inter-Observer Agreement. Bland Altman plots for inter-observer agreement of left ventricular **a**) end-diastolic volume (EDV), **b**) end-systolic volume (ESV), **c**) stroke volume (SV) and **d**) ejection fraction (EF). Continues black lines represent bias and dashed lines indicate 95% limits of agreement

Inter-observer variability assessed in the 15 volumetric data sets revealed good agreement with standard deviations of differences of 3.2% (bias − 0.4%; 95% limits of agreement − 6.6% – 5.8%) for EDV, 3.3% (bias 0.2%; 95% limits of agreement − 6.2 – 6.6%) for ESV, 5.8% (bias − 0.4%; 95% limits of agreement − 11.8 – 11%) for SV, and 2.9% (bias 0%; 95% limits of agreement − 5.7 – 5.7%) for EF. Assessment of myocardial wall thickness in end-diastole demonstrated similar results with standard deviations of differences between single measurements of 4.5% (bias − 1.7%; 95% limits of agreement − 10.6% - 7.1%). Inter-observer variability of volumetric measurements is illustrated as Bland Altman analysis (Fig. 6**)**.

## Discussion

This study demonstrates the successful application of a newly developed DUS device for gated fetal CMR imaging. Dynamic CMR was performed using an external sensor to track the fetal cardiac cycle. In this multicenter study, dynamic cardiac-gated images were acquired in all 15 fetuses with a gestational age range of 33–39 weeks. The DUS device caused no image artifacts and no complications. Further, none of the pregnant women complained about the transducer or aborted the scan due to the device. The incomplete exams in 4 pregnant women were due to discomfort related to the small confines of than scanner bore. In some cases the extent of the fetal cardiac examination was limited by loss of the DUS gating signal related to major fetal motion and maternal breath-holding. Image quality was rated moderate to high and allowed reliable identification of fetal cardiac structures. The myocardial border sharpness was quantified by measuring EB and demonstrated good results with a reliable assessment of volumetric data. This technique has the potential to improve the effectiveness of fetal CMR and to yield novel information about cardiac function and blood flow in the developing fetus.

The results of the current study demonstrate successful application of the DUS transducer for gating fetal CMR. The transducer was not influenced by the CMR scanner and image quality was not affected. The generated DUS signal used for fetal cardiac gating showed a sensitivity of 96% for cardiac cycle recognition, which is similar to reported sensitivities of ECG gating in adults [17, 18]. Moreover, one indicator for correct detection of the cardiac cycle is the variability of the gating signal. The measured heart rate variability of 26 ms is similar to previously reported values ranging from 16 to 25 ms,.suggesting reliable detection of each heart cycle for freezing cardiac motion without image blurring. However, at this point it must be mentioned that the variability of the gating signal is rather an indicator than a quantitative measure. The capability of DUS gating to freeze cardiac motion was further quantified by determination of EB. EB of fetal dynamic cardiac short-axis images (2.7 ± 0.8 pixels) was similar to adult values (2.6–3.3 pixels) using DUS gating [8, 9]. The observation of lower end-diastolic EB can be explained by reduced radial, tangential, and longitudinal myocardial velocities during diastole, and is in agreement to findings of DUS gated CMR in adults [8]. Both the high image quality and little impact of motion blurring with clear differentiation between myocardium and lumen indicate high gating quality. In this work a temporal resolution of 14–24 ms was used to capture fetal cardiac wall motion to assess fetal cardiac function. In prior work by Haris et al. and Roy et al. fetal cardiac function was assessed with a temporal resolution ranging from 31 to 40 ms [14, 19]. As the temporal resolution was even lower in this study, we assume it is sufficient to capture fetal cardiac wall motion. However, it must be mentioned that there is only limited experience in dynamic fetal CMR and thus no established reference CMR protocols so far.

In the current study dynamic fetal CMR and morphological analysis was focused on the 4-chamber view as this is commonly used for evaluation of fetal cardiac pathology, with sensitivity and specificity of 88% and 96%, respectively [20, 21]. The possibility to synchronize the CMR data acquisition to the fetal heart beat offers high image quality for visualizing small structures. In the current study the atrial septum was visualized in all cases, whereas a previous study using non-gated bSSFP sequences only managed this in one third of the fetuses [22]. Also, the foramen ovale was visualized in all fetuses in the current study but varied widely in previous studies, between 6% using real-time cine imaging [23] and 95% using non-gated bSSFP images [24]. Myocardial wall thickness was comparable to recently published data using metric optimized gating (MOG) (2.6 ± 0.3 mm), self-gating (2.7 ± 0.3 mm) and real time imaging (3.0 ± 0.4 mm) [14]. The slightly higher values in our study (3.4 ± 0.5 mm) may be due to methodological differences as measurements in our study were assessed from the midcardial septum instead from the postero-lateral LV wall. In addition, the assessed LV stroke volumes of 1.9 (± 0.8) ml in our study subjects with gestational ages ranging from 30 to 39 weeks are comparable with reported ultrasound data of 2.1 ml (95% CI 1.27, 3.4) for fetuses of 34 weeks gestational age [25]. The mean EDV of 2.8 (± 1.2) ml calculated for the fetuses in this study are also very similar to the EDV of a large fetal ultrasound study [26]. Furthermore, the relatively high standard deviation of assessed EDV in this study is in concordance of the wide range of EDV between 30 and 39 weeks of gestation, approximately ranging form 0.5–5.5 ml [26].

Until now fetal CMR has been limited due to technical challenges [4], mainly the lack of a fetal ECG for gating. As a consequence of unavailable direct fetal cardiac gating so far, dynamic fetal CMR was dependent on complicated post-processing techniques only available in a few centers. A major innovation was the introduction of MOG [27]. Image data is continuously acquired without gating and iteratively reconstructed at varying heart rates. A gating signal is determined retrospectively by optimizing an image metric [19, 28]. Several studies proved feasibility of this technique performing phase-contrast angiography for the assessment of fetal blood flow characteristics including 3 T application [29–32]. A clear limitation of this method is that MOG is prone to heart rate variability, namely the inability to adjust the gating signal to varying heart frequencies. Therefore, the modeled fetal heart rates may be a potential source of error when performing quantitative measurements. This aspect has to be emphasized considering the broad variation of fetal heart rate also in the healthy fetus, with reported frequencies between 138 and 175 bpm during CMR examination [27]. Post-processing of the acquired MOG images can also be extensive, and requires specialized software. Depending on the processing algorithm, reconstruction times from 10 min up to 2 h for a single slice were reported [28]. Although MOG can produce high-quality diagnostic images the post-processing times illustrate the limitation of all such techniques for widespread implementation in clinical routine.

Another approach to realize fetal cardiac gating is the self-gating method. This technique estimates a periodic gating signal by processing acquired k-space data [33]. A fully sampled k-space data set is then fed into a reconstruction algorithm [34]. The self-gating method identifies characteristic patterns of the cardiac cycle (e.g. contraction) that are used as reference for the following cardiac cycles [35]. Studies in fetal sheep revealed satisfactory image quality that was only slightly inferior to that of pulse-wave triggered images [36]. Feasibility of high-quality functional imaging of small sized hearts using self-gating was demonstrated in rats at 3 T [37]. Only recently self-gated CMR of the human fetus using parallel imaging and sparse sampling modifications (iGRASP) to accelerate image acquisition was successfully performed. The study demonstrated equal results for quantitative and qualitative measures comparing self-gating and MOG, however with improved image quality [14]. Albeit reconstruction now can be performed in a matter of minutes the technique is dependent on specialized software.

Although MOG and self-gating methods can provide high-resolution images, the post-processing steps are time-consuming and preclude rapid reconstruction; consequently, there is little opportunity to review data quality during the examination or adapt imaging planes in case of fetal movement. Whereas fetal echocardiography represents a fast, non-invasive and widely available diagnostic tool allowing for real-time imaging with high spatial resolution [38], improved fetal CMR with gated acquisition may be crucial in selected cases for enhanced diagnostic quality.

A limitation of the current study is the focus on 4-chamber and short-axis views only. However, this initial step is sufficient for improved utility of fetal CMR imaging as the 4-chamber view is commonly used for diagnosis of fetal cardiac malformation. Future studies have to evaluate DUS gated fetal CMR imaging for other imaging planes and also other anatomic structures, also potentially including reference-imaging standards for comparison. Finally, fetal movement during CMR may require re-positioning of the DUS transducer with prolonged examination times, however already indicated to not be a major issue. Moreover, fetal movement or uterus contractions may lead to disrupted gating signals. A potential enhancement of the DUS gating method would be a wider ultrasound field, covering a larger portion of the maternal abdomen to prevent repositioning of the DUS transducer in case of major fetal movement. Finally, there was only a small study population that was divided over 3 centers using different scan parameters. For example, the protocols differed in the coverage of heart phases (30 phases to 20 phases), which could have affected EB analysis.

## Conclusion

High-quality dynamic fetal CMR was successfully performed for the first time in human fetuses using a newly developed DUS device for direct cardiac gating. This technique has high potential to improve the utility of fetal CMR in the evaluation of congenital pathologies.

## Abbreviations

BNC: Bayonet Neill Concelman
bSSFP: balanced stead state free precession
CMR: cardiovascular magnetic resonance
DUS: Doppler Ultrasound
EB: endocardial blurring
EBS: endocardial border sharpness
EDV: end-diastolic volume
EF: ejection fraction
ESV: end-systolic volume
LV: left ventricular/left ventricle
MOG: metric optimized gating
RF: radiofrequency
SV: stroke volume
TTL: transistor-transistor-logic
